# Surgical approach on combined chronic patellar tendon and bicruciate knee ligament injury

**DOI:** 10.1186/s13018-024-04724-w

**Published:** 2024-05-28

**Authors:** Sérgio Rocha Piedade, Carlos Górios, Filippo Spiezia, Nicola Maffulli

**Affiliations:** 1 Exercise and Sports Medicine, Department of Orthopaedic, Rheumatology, and Traumatology, School of Medical Sciences, University of Campinas, UNICAMP, Campinas, Brazil; 2 Centro Universitário São Camilo, Ipiranga, São Paulo Brazil; 3 Department of Science, Basilicata University, UNIBAS, Potenza, Italy; 4 Department of Orthopaedic and Trauma Surgery, Ospedale San Carlo, Potenza, Basilicata, Italy; 5 Department of Orthopaedics and Traumatology, Faculty of Medicine and Surgery, Surgery and Dentistry, Sapienza University, Roma, 00100 Italy; 6 Centre for Sports and Exercise Medicine, Barts and The London School of Medicine and Dentistry, Mile End Hospital, Queen Mary University of London, London, E1 4DG UK; 7 School of Pharmacy and Bioengineering, Keele University School of Medicine, Thornburrow Drive, Stoke On Trent, England

**Keywords:** Knee injuries; patellar ligament: transplants, Posterior cruciate ligament, Anterior cruciate ligament

## Abstract

A combined injury of the patellar tendon and both the anterior and posterior cruciate ligaments is disabling. It directly affects knee kinematics and biomechanics, presenting a considerable surgical challenge. In this complex and uncommon injury, decision-making should take into account the surgeon’s experience and consider one- or two-stage surgery, tendon graft, graft fixation, and rehabilitation protocol. This manuscript discusses the surgical approach based on a comprehensive understanding of the patellar tendon and bicruciate biomechanics to guide which structures should be reconstructed first, especially when a two-stage procedure is chosen.

## Introduction

A combined injury of the patellar tendon and both the anterior and posterior cruciate ligaments is disabling. It directly affects knee kinematics and biomechanics, presenting a considerable surgical challenge [1, 2].

Although surgery is the treatment of choice, decision-making should take into account the surgeon’s experience and consider one- or two-stage surgery, tendon graft, graft fixation, and rehabilitation protocol [3–6]. Moreover, a comprehensive understanding of the patellar tendon and bicruciate biomechanics is vital to guide which structures should be reconstructed first, especially when a two-stage procedure is chosen [7, 8].

In multi-ligament knee injuries, allografts may reduce the duration of the procedure, allowing an unlimited number of grafts and different graft sizes [9, 10]. However, they may not be readily or widely available, and some surgeons may not be familiar with them. On the other hand, autografts optimize biological integration, but, their number and availability may be limited, and the morbidity associated with their harvest should be taken into account [10, 11].

In knee surgery, posterior cruciate ligament (PCL) reconstruction can be accomplished using the transtibial tunnel or tibial inlay technique. Both methods are supported by published evidence [12, 13].

This manuscript discusses management strategies for a combined chronic injury of the patellar tendon and both the anterior and posterior cruciate ligaments.

## Case presentation

**Fig. 1:**
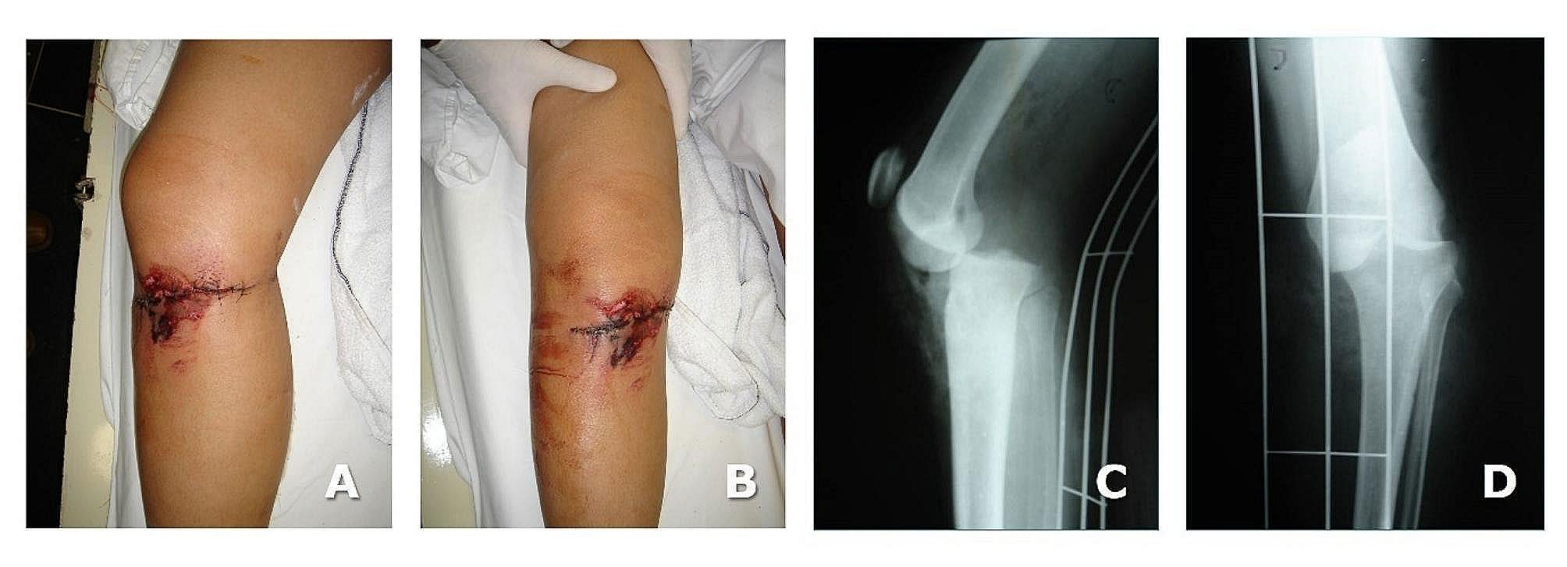
Clinical appearance of the right knee, sutured skin laceration on the day of injury (**A** and **B**), and (**C**) lateral and (**D**) anteroposterior radiographs

A 21-year-old woman was involved in a motorcycle accident and suffered a combined patellar tendon, anterior cruciate ligament (ACL), posterior cruciate ligament (PCL) in her knee. She presented a skin laceration over the proximal anteromedial aspect of the right knee: this was washed out and sutured at her local Accident and Emergency Department (Fig. 1A, B, C, **and D**). According to the patient, no actual diagnosis was formulated, and she missed the planned clinical follow-up for personal and economic reasons.

**Fig. 2:**
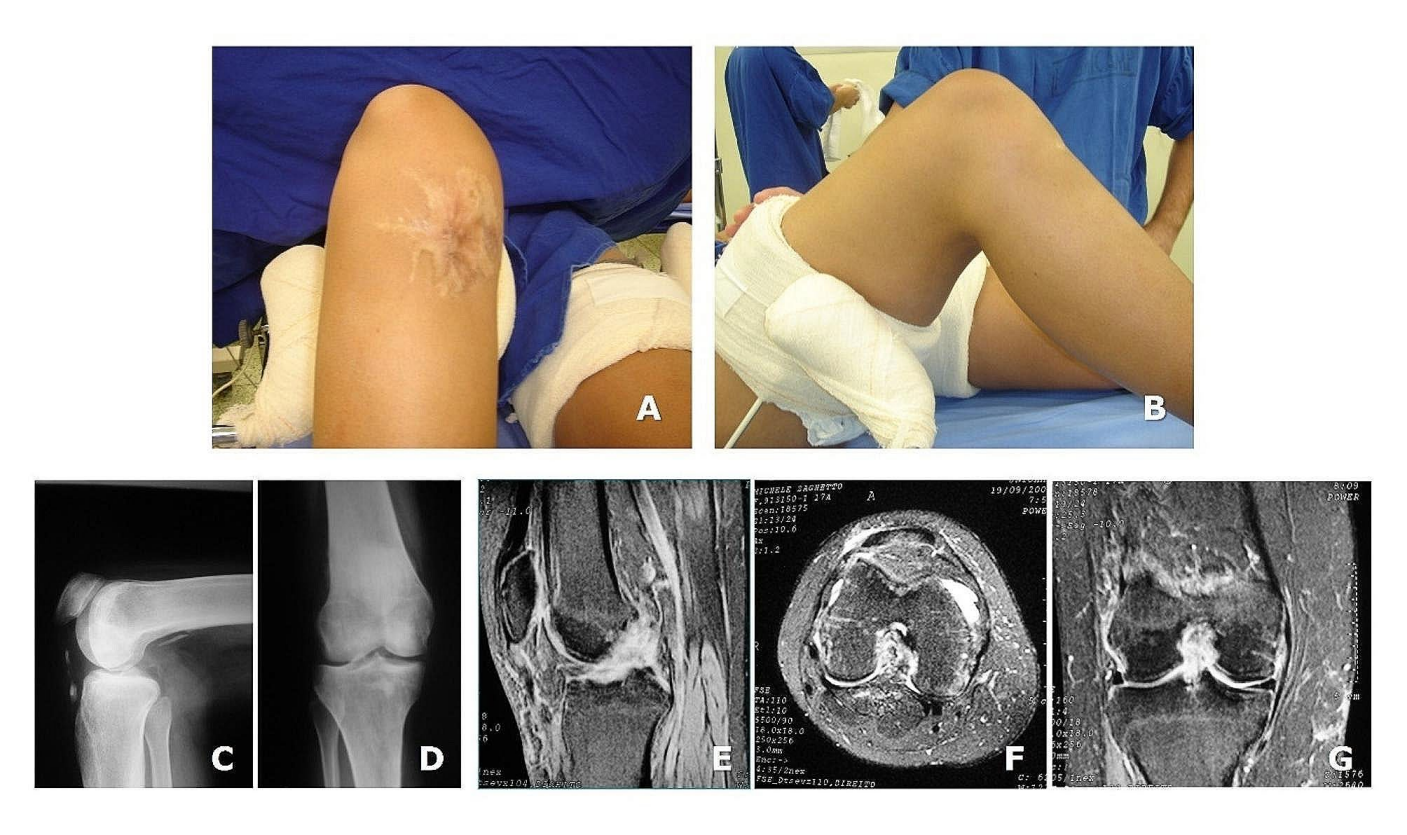
One year after the accident, on physical examination a well-healed scar (**A**) and posterior sag sign (**B**), indicating PCL insufficiency, Panels **C** to **G**: Imaging appearance (**C** and D: plain radiographs); **E** to **G**: sagittal, axial, and coronal MRI scans. They demonstrate the chronic patellar tendon rupture and the tear of both the anterior and posterior cruciate ligaments, a chondral Injury in the medial femoral condyle, a grade-II medial collateral ligament injury, and a minor medial meniscus injury, with no ligament injury of the lateral knee compartment, as also confirmed during the surgical procedure

One year after the accident, the patient attended the hospital again, and a careful physical examination, plain radiographs, and an MRI assessment were performed. The physical examination showed a patella alta and inability to fully extend the knee actively, grade 3 posterior laxity, and grade 2 anterior laxity, and the lateral and medial ligament compartments did not present pathological laxity. The chronic patellar tendon rupture makes clinical assessment difficult. Therefore, the diagnosis of a combined chronic patellar tendon and ACL and PCL ruptures was formulated. (Fig. 2A, B, C, D, E, F **and G**).

The knee injuries were managed surgically by a two-stage surgical procedure, starting with a concomitant patellar tendon and PCL tibial inlay reconstruction. The knee was left in full extension brace for the first six postoperative weeks. Knee mobilization started at the end of this period after brace removal.

The ACL reconstruction was performed three months after the first procedure, when more than 100 degrees of active knee flexion were achieved. During surgery, the wire and the cortical screw used to protect the patellar tendon graft were removed. Next, the ipsilateral tendons of semitendinosus and gracilis were harvested to perform standard four-strand ACL reconstruction, fixed by a cross pin on the femoral side and washer-lock and screw on the tibial side. Then, rehabilitation followed, as standard ACL reconstruction.

### First STAGE of Surgical approach (part one) - patient in dorsal decubitus

**Fig. 3:**
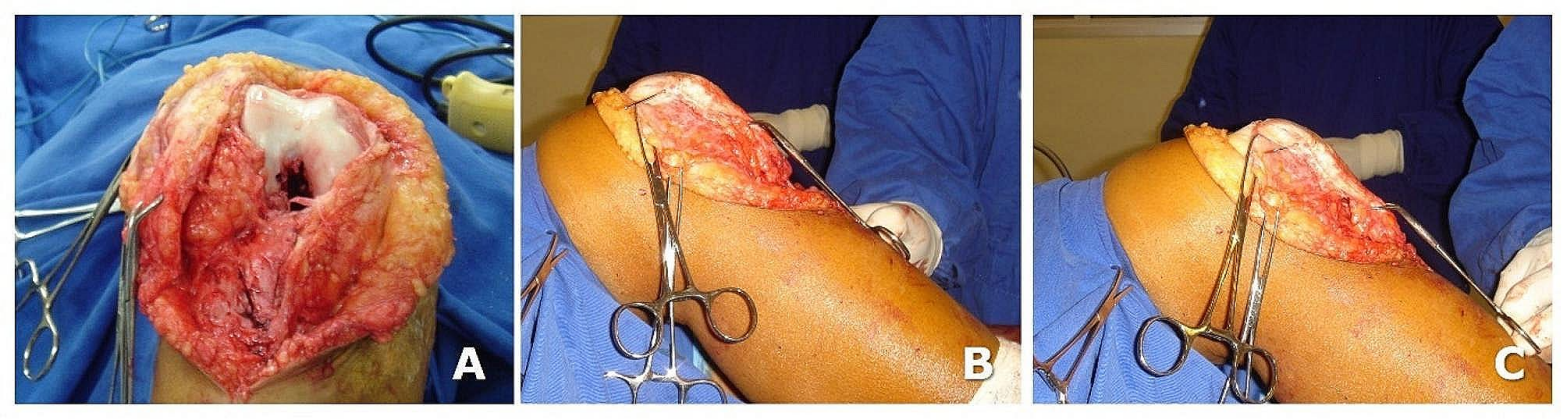
Intraoperative view. (**A**) the empty intercondylar notch indicates bicruciate ligament injury. **B** and **C**: determining and adjusting the patella height using a Kirschner wire as reference

With the patient positioned supine, midline skin incision, a 12 cm approach allowed to expose the knee joint to assess the avulsion of the patellar tendon from its distal insertion, the height of the patella, and the intercondylar notch. (Fig. 3A, B, **and C**). The bone patellar tendon (BTB) graft and hamstring grafts were harvested from the contralateral knee, and the harvest site sutured.

We routinely use an OUT-IN guide to drill the PCL femoral tunnel. However, as in this patient, the chronic patellar tendon tear offered a wide exposure of the femur (Fig. 3A), we performed an 8 mm PCL femoral tunnel using an IN-OUT guide to accommodate the BTB graft.

**Fig. 4:**
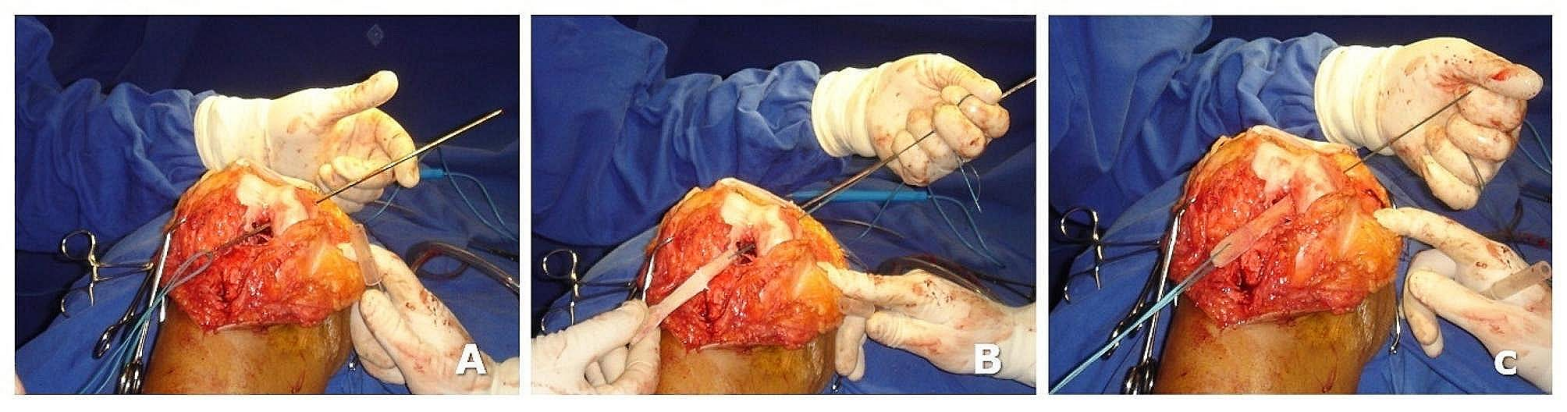
Stages of PCL tibial inlay reconstruction on the femoral side with PCL femoral tunnel and graft passage and fixation with a femoral interference screw with the patient supine: (**A**, **B**, and **C**) graft passage

**Fig. 5:**
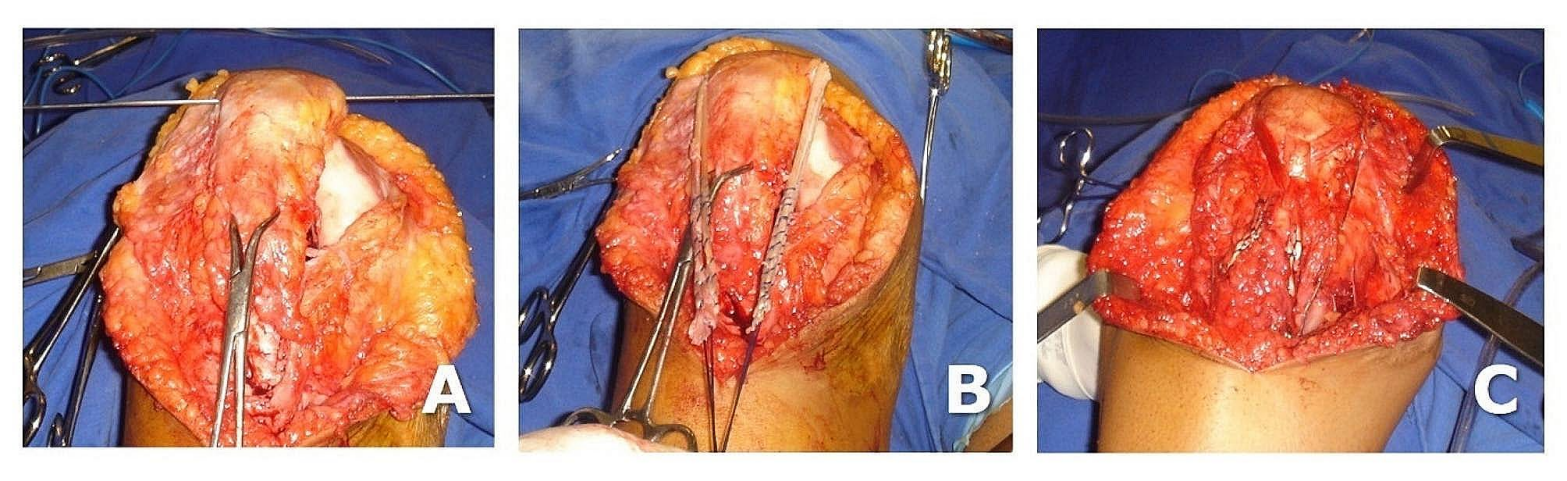
Intraoperative view of concomitant surgical reconstructions of the patellar tendon using hamstring grafts harvested from the contralateral (left) knee (**A**, **B**, and **C**)

Then, the graft was passed into the PCL femoral tunnel and fixed using an 8 mm interference screw. The free end of the graft was positioned intra-articularly with a 5.0 Ethibond suture passed into the bone block (Figs. 4 and 5).

**Fig. 6:**
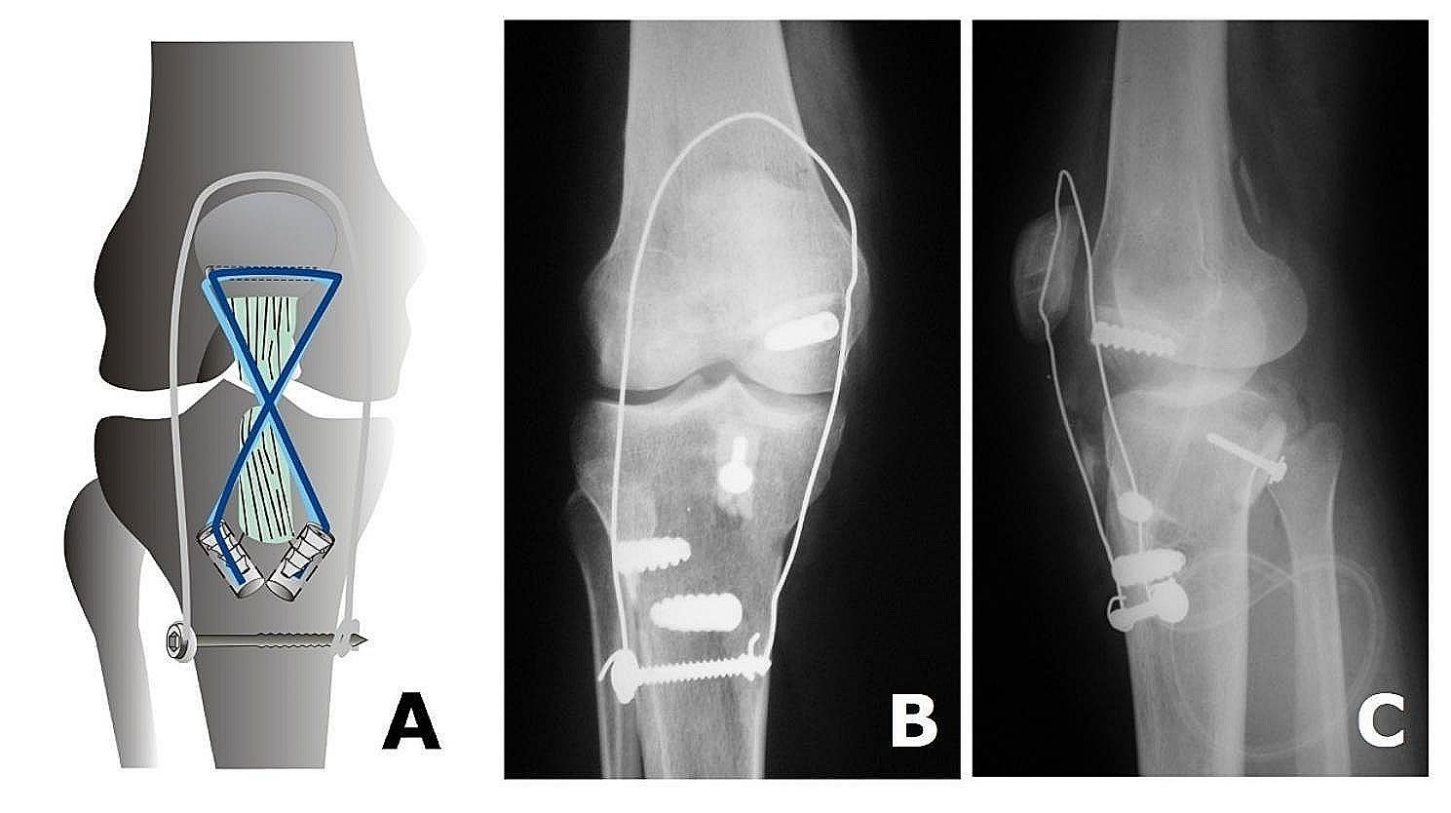
The patellar tendon and PCL reconstructions (**A**) and postoperative radiographs ((A) anteroposterior and lateral views (**B**))

Concomitantly to PCL tibial tunnel BTB graft fixation, the patellar tendon was reconstructed. The remaining tissue of the free end of PT was too short and had an associated scar tissue that did not allow it to be re-inserted in the tibial site of the patellar original insertion. Therefore, we used the semitendinosus and gracilis tendons harvested from the contralateral knee, passing through a transverse tunnel in the patella. Then, the graft was directly distally and crosses through the remaining PT tendon, and fixed on the tibia after manually reducing the knee posterior sag using two interference screws and protecting the graft by using a metal wire and cortical screw on the tibia (Figs 5 and 6). Finally, the wound was sutured in standard fashion.

### Second stage of the First Surgical approach - patient in ventral decubitus

#### PCL and patellar tendon reconstruction

**Fig. 7:**
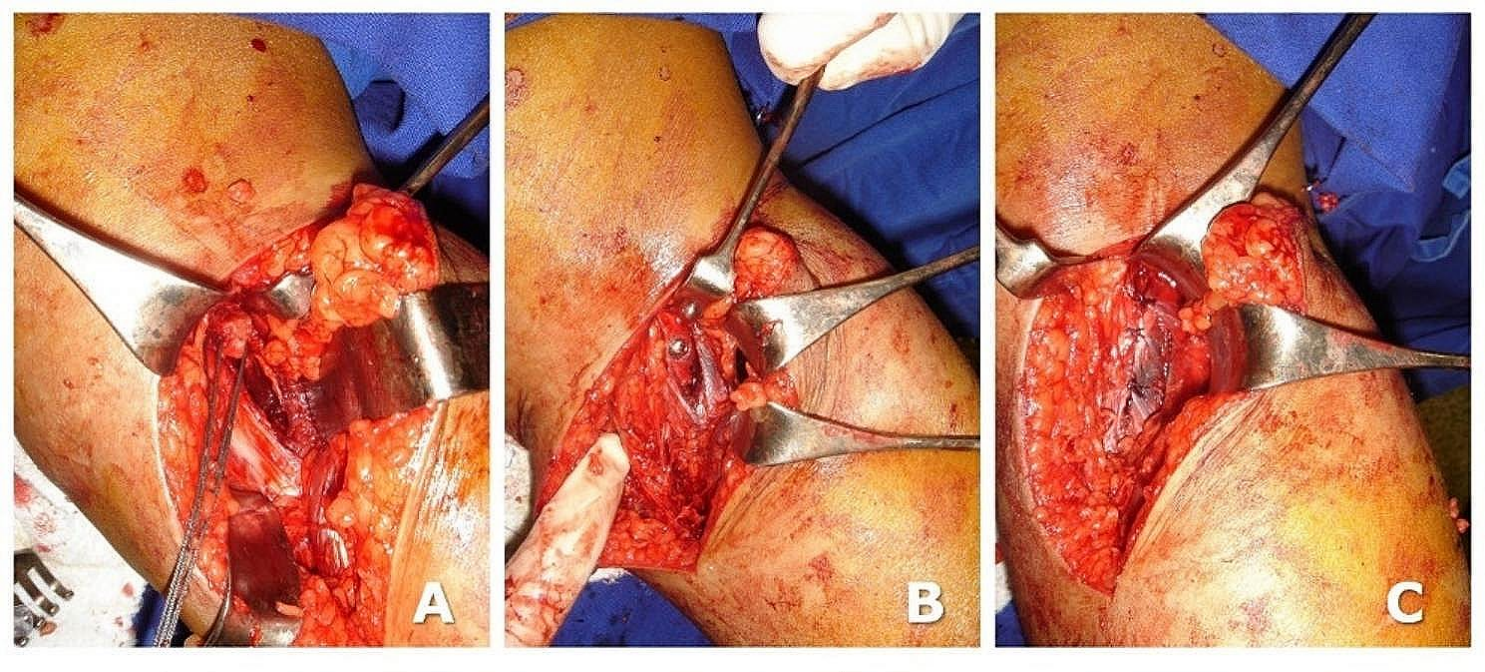
PCL tibial inlay reconstruction on the tibial side: (**A**) recovering the free end of the BTB graft and producing the PCL tibial slot, (**B**) fixing the bone graft fixation by bone press-fit, pull-out, and 3.5 mm cortical screw. (**C**) capsule suture

The patient was positioned prone, and an inverted “L” posteromedial incision was performed, with the horizontal branch of the L placed in the knee flexion crease. Then, the deep fascia was resected vertically. A blunt dissection between the medial border of the gastrocnemius muscle and the semimembranosus tendons was performed. The medial edge of the gastrocnemius was retracted laterally and posteriorly to expose the posterior capsule, which was opened. The free end of the BTB graft was attached to a 5.0 Ethibond suture to be recovered. Using custom made instruments, we produced a tibial slot (10 mm long, 9 mm wide, and 10 mm deep) at the PCL tibial insertion [14]. The free end of the BTB graft was placed on the tibial slot and fixed using a bone pull-out press-fit 3.5 mm cortical screw and a washer, and the capsule sutured with the knee kept in full extension (Fig. 7).

## Second Surgical approach

The ACL reconstruction was performed three months after the first operation when knee flexion greater than 110° flexion was obtained.

### ACL reconstruction

With the patient supine and under spinal anesthesia, the knee was kept flexed at 90° using a thigh tourniquet.

**Fig. 8:**
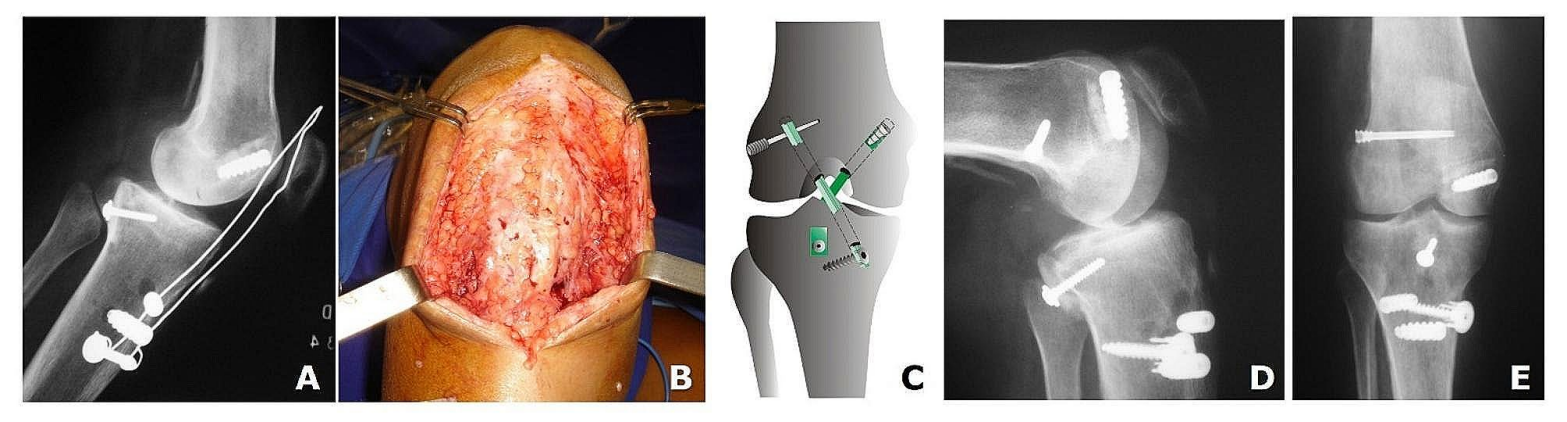
(**A**) Postoperative radiographs (lateral view) of the right knee, (**B**) Intraoperative view after removing the wire and screw of patellar tendon reconstruction: the hamstring graft was fully integrated into the patellar tendon, (**C**) schematic drawing of technique for bicruciate knee ligament reconstruction. ((**D**) lateral and (**E**) anteroposterior postoperative radiographs

A midline incision was performed, and the wire and screw used to protect the patellar tendon graft were removed (Fig. 8-A and B).

Then, the ACL femoral tunnel was drilled using an outside-in technique, under arthroscopic control. Initially, the bone tunnel was drilled according to the diameter of the harvested graft (6 mm). The gracilis and semitendinosus tendons of the ipsilateral knee were harvested.

A tibial tunnel 6 mm in diameter was drilled at a 55° angle using a tibial guide under direct arthroscopic control. Again, the tunnel diameter was adjusted after measuring the four-strand graft (double semitendinosus and double gracilis). After drilling all bone tunnels, the length of the ACL was measured, and the graft prepared with a Vicryl 2.0 suture.

The graft was then shuttled into the knee joint through the tunnels. The graft was fixed on the femoral side using a cross pin system. The knee was then flexed and extended 20 times to tension the graft, followed by fixation on the tibial side using a screw with a washer-lock system (Fig. 8-C, D and, E).

The anteroposterior stability of the knee was tested and confirmed using the Lachman test, and the knee range of motion was checked. The tourniquet was released, accurate hemostasis was performed, and the wound was sutured. The knee was bandaged in a standard fashion, and weight-bearing was allowed and encouraged after recovering from anesthesia. The patient used two crutches only for the first two weeks, and rehabilitation followed the protocol as for primary ACL reconstruction [15].

### Rehabilitation protocol

**T**he rehabilitation plan was defined preoperatively after discussion with the physiotherapy team. We stressed that the surgical plan involved a staged procedure, which started with a simultaneous reconstruction of the primary (PCL) and secondary restrictor (PT) to tibial posterior translation. The second stage was ACL reconstruction, performed when the knee flexion was at least 100°.

Following the first procedure, the knee was kept in full extension for the first six weeks, and weight-bearing was allowed using crutches. Rehabilitation focused on pain control, anti-inflammatory measures, and isometric contraction of the quadriceps muscle complex, under physiotherapist supervision, restricting the passive knee flexion until 30 degrees. After the sixth week, we aimed to reactivate and strengthen the knee extensor mechanism to help and protect the PCL tendon graft, for the first three months postoperatively. Knee flexion was performed with the patient prone.

At three months postoperatively knee flexion reached 100°, and ACL reconstruction was carried out. The rehabilitation protocol at this stage followed the same routine of an ordinary ACL: analgesia, reducing swelling and inflammation, weight-bearing starting on the second postoperative day using crutches for two weeks, reestablishing full ROM and neuromotor control of the knee, strengthing of quadriceps and hamstrings, and return to daily and physical activities [15]. The patient had a moderate level of physical activity, mainly jogging.

### Outcomes

At 7 months postoperatively, the patient progressively returned to her daily physical activities, and rehabilitation was discontinued. The patient was asked to keep doing exercises for quadriceps strengthing and motor control at home.

At three years of follow-up, the patient returned to her daily work activities and progressed without restrictions, but it is essential to consider that she has **moderate physical activity. The physical assessment recorded** a full range of motion (Fig. 10A, B, and C), and the posterior draw reduced from 3 + to 1+, with no sign of undie laxity of the lateral and medial collateral ligaments.

The patient had returned to jogging and general calisthenics.

**Fig. 9:**
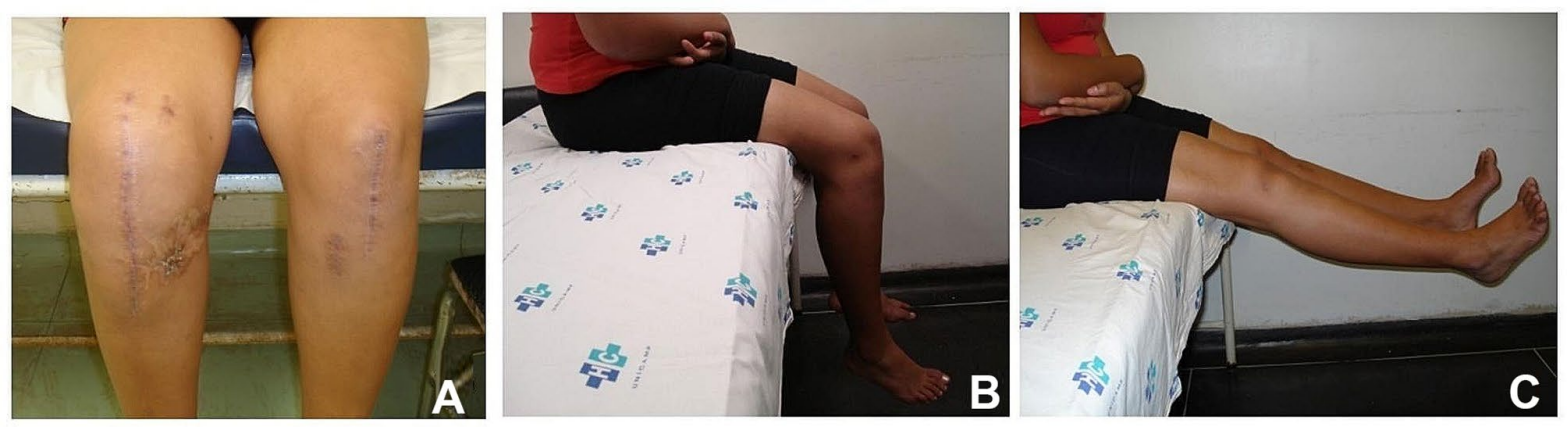
Clinical presentation of knees (**A**) scars and (**B** and **C**) active knee extension

## Discussion

Combined patellar tendon and bicruciate ligaments rupture is an uncommon injury, resulting from high-energy trauma and involving tendon damage to the knee extensor complex (patellar tendon) and both cruciate ligaments [6, 16].

In general, multiple ligament injuries produce major knee instability and negatively impact the patient’s quality of life. The management is surgical and should address all injured structures, a true challenge for both surgeon and patient [9, 17].

Being a complex knee ligament injury, surgical strategies may vary; one should consider single or a staged procedure, tendon graft option, using allograft, or even synthetic ligament [17, 18]. However, this scenario can be still more challenging facing chronic injuries, requiring a unique and reliable treatment strategy. The surgical plan should be defined by assessing each patient on their own merit, and also taking into account the surgeon’s experience: familiarity with these complex injuries remains the key to decision-making.

A single procedure can be advantageous for the surgeon and the patient. However, this is a rare injury and unfamiliar to many surgeons. Its approach involves technical challenges, including the surgeon’s experience, the unavailability of allografts and, according to the surgical approach adopted, the need to change the patient’s position during the procedure; these must be taken into consideration.

A genuine concern approaching complex knee ligament injuries is the local tissue trauma, mainly when using autografts, as it adds further morbidity to the surgery. In this context, knee stiffness, infection, and skin necrosis are possible complications, especially in the presence of a previous scar on the knee. Therefore, preoperative evaluation should take into account all of these potential problems, and a staged surgery could well be chosen [19, 20].

A genuine concern approaching complex knee ligament injuries is the local tissue trauma, mainly when using autografts, as it adds further morbidity to the surgery. In this context, knee stiffness, infection, and skin necrosis are possible complications, especially in the presence of a previous scar on the knee [21, 22].

Therefore, preoperative evaluation should take into account all of these potential problems, and a staged surgery could well be chosen.

When approaching these complex injuries, the primary strategy is to minimize additional tissue damage, even in a chronic injury. Over the last 20 years, after changing to PCL inlay reconstruction, we observed only minor knee effusions in our clinical practice, and the gain of knee range of movement was greater compared to the PCL tibial tunnel technique [14]. Hence, inlay reconstructions become our preferred technique to reconstruct the PCL.

A chronic patellar tendon tear may not be directly repaired, given the gap at the injury site, usually filled out by scar tissue [23]. Therefore, tendon graft augmentation is recommended, using an autograft or allograft [10]. Furthermore, the procedure should restore the appropriate patellar height, paying attention to protecting graft integration and sheltering the graft from undue tension, particularly in the early postoperative months [23–26].

Regarding the patellar tendon reconstruction, at the time of the first procedure, we used two interference screws to fix the tendon graft and protected the graft by using a metal wire and cortical screw on the tibia. At present, we use only one interference screw on the tibia tunnel and no metal wire and cortical screw to protect the graft.

Similarly, a chronic bicruciate knee ligament injury is technically demanding, even in experienced hands, as it involves the reconstruction of two ligaments, with two or more tendon grafts, choosing the graft fixation system, and using different surgical techniques [27, 28].

When managing a combined chronic patellar tendon and bicruciate knee ligament injury, using allografts may reduce the duration of surgery [10].

Decision-making should be based on careful physical examination combined with in-depth knowledge of the function of each injured ligament, the knee’s biomechanics and kinematics, which are crucial for the surgeon to define the correct management [4, 5, 9, 20, 28].

For both single and staged surgery, PCL reconstruction plays a pivotal role in restoring the appropriate anatomical relationship of the knee joint surfaces, and, therefore, it should be reconstructed first independently of the following surgical options [5, 20, 27, 28].

At three years postoperative clinical assessment, the knee stability had substantially improved to a residual 1 + posterior drawer, with full active knee extension. Moreover, the patient returned to her usual physical activities, including jogging and other physical activities, a successful outcome for such chronic multi-ligament knee injury managed through a two-stage surgical procedure.

This may suggest that reconstruction of PCL and PT may play a reciprocal protective effect after deleterious overstretching of grafts.

## Conclusion

As with any complex knee ligament injury, surgical strategies may vary; surgeons should consider single or a staged surgery, tendon graft options, using allografts, or even synthetic ligaments. The procedure performed in our patient has been successful, but we are aware that no level I studies favor one approach over another. However, we recommend that such patients come under the care of experienced knee surgeons, given the technical demands of these injuries.

## Data Availability

No datasets were generated or analysed during the current study.
